# IMPACT OF UNIFORMS IN LONG-TERM CARE FACILITIES: SYNTHESIS OF INTERNATIONAL EVIDENCE AND SURVEY OF UK STAFF VIEWS

**DOI:** 10.1093/geroni/igad104.2291

**Published:** 2023-12-21

**Authors:** Jennifer Liddle, Katie Thomson, Abdulwahab Al-Azawi, Dawn Esslemont, Barbara Hanratty, Christine Henderson, Louise Jones, Lisa Wilson

**Affiliations:** Newcastle University, Newcastle upon Tyne, England, United Kingdom; Newcastle University, Newcastle upon Tyne, England, United Kingdom; Newcastle University, Newcastle upon Tyne, England, United Kingdom; Eothen Care Homes, Cramlington, England, United Kingdom; Newcastle University, Newcastle upon Tyne, England, United Kingdom; Eothen Care Homes, Wallsend, England, United Kingdom; Northumbria Healthcare NHS Foundation Trust, Ashington, England, United Kingdom; Eothen Care Homes, Whitley Bay, England, United Kingdom

## Abstract

Non-uniform-based staff dress codes are beginning to emerge in UK long-term care facilities for older people. However, there is little research evidence on the impact of staff clothing on resident, staff and visitor experiences, wellbeing or quality of life. Uniforms may be reassuring and facilitate identification of staff; a non-uniform approach may create a home-like, relaxed environment. This study aimed to synthesise existing international evidence and explore staff views about work clothing in three not-for-profit long-term care facilities in the UK. Five bibliometric databases were searched from inception. A narrative synthesis of included papers was conducted due to heterogeneity of study designs and outcome reporting. 77 (49%) of 158 staff responded to the survey. 68 staff (88%) did not currently wear a uniform at work. Views about the importance and impact of uniform were mixed: 25 (32%) believed that their role needed a uniform; 60 (78%) thought staff clothing made no difference in resident care quality but 45 (58%) agreed the environment felt more home-like when staff did not wear a uniform. 36 staff (47%) would choose to wear a uniform and 17 staff (22%) reported finding it difficult to afford clothes for work. In the context of limited research evidence, staff clothing is perceived to have little impact on care quality but considerable impact on long-term care facility atmosphere and staff working experiences. There is a need to research the impact of staff clothing to support evidence-based decisions about staff clothing policies in long-term care settings.

